# Supplementary material for: Content-rich biological network constructed by mining PubMed abstracts
Source: BMC Bioinformatics. 2004 Oct 8;5:147. doi: 10.1186/1471-2105-5-147 (PMC528731; doi:10.1186/1471-2105-5-147)
Supplement: Additional File 5 — The original Chilibot query results of the term "long-term potentiation (LTP)" and 22 other terms, limiting the latest references analyzed to the years 1990, 1995, 2000, and 2004. [file 1471-2105-5-147-S5.bz2 › chilibotAdditionalFile5/ltp2004/html/PKA_ACTININ.html]

 


 **PKA** and **ACTININ** 
  
Found 3 abstracts in PubMed,  **3 abstracts were retrieved and analyzed**.  


---

 Search Google  |
 PDF files only 
|  EDU domain only 

---

- Mol Pharmacol, 2002   The regulation of cardiac delayed rectifier potassium currents by cAMP dependent protein kinase contributes to the control of blood pressure and heart rate.
  We investigated the modulation by **PKA** and protein phosphatases of cloned Kv1.5 channels expressed in Xenopus laevis oocytes.
  Exposure of oocytes to activators of **PKA** had no effect on the amplitude of Kv1.5 currents.
  Inhibition of **PKA** by injection of protein kinase A inhibitor peptide or exposure to myristoylated protein kinase A inhibitor peptide reduced currents mediated by Kv1.5.
  M PKI also reduced the amplitude of currents mediated by mutated Kv1.5 channels in which the COOH terminal **PKA** phosphorylation sites and PSD 95, Disc large, and ZO 1 binding domain were removed.
  The reduction of Kv1.5 currents by M PKI was attenuated by inhibition of actin polymerization by 1 microM cytochalasins B and D, but was not affected by 10 microM phalloidin or 50 microM colchicine .
  Treatment of oocytes with antisense oligonucleotides against alpha **actinin** 2 abolished the reduction in Kv1.5 current by M PKI.
  These observations suggest that Kv1.5 currents are activated by endogenous **PKA** in resting oocytes.
  inhibition of **PKA** activity reveals the action of endogenous phosphatases.
  Indeed, injection of alkaline phosphatase reduced currents mediated by Kv1.5.
  Further preincubation of oocytes with 1 mM sodium orthovanadate abolished the reduction in Kv1.5 currents by M PKI.
  We conclude that currents encoded by Kv1.5 are regulated by **PKA** and protein tyrosine phosphatase.
  this regulation requires an intact actin cytoskeleton and alpha **actinin** 2.

  - J Cell Biol, 1998   **A kinase anchoring protein 100 AKAP100 is localized in multiple subcellular compartments in the adult rat heart.** .
    Stimulation of beta adrenergic receptors activates type I and II cyclic AMP dependent protein kinase A, resulting in phosphorylation of various proteins in the heart.
    It has been proposed that **PKA** II compartmentalization by A kinase anchoring proteins AKAPs regulates cyclic AMP dependent signaling in the cell.
    We investigated the expression and localization of AKAP100 in adult hearts.
    By immunoblotting, we identified AKAP100 in adult rat and human hearts, and showed that type I and II regulatory RI and II subunits of **PKA** are present in the rat heart.
    By immunofluorescence and confocal microscopy of rat cardiac myocytes and cryostat sections of rat left ventricle papillary muscles, we localized AKAP100 to the nucleus, sarcolemma, intercalated disc, and at the level of the Z line.
    After double immunostaining of transverse cross sections of the papillary muscles with AKAP100 plus alpha **actinin** specific antibodies or AKAP100 plus ryanodine receptor specific antibodies, confocal images showed AKAP100 localization at the region of the transverse tubule junctional sarcoplasmic reticulum.
    RI is distributed differently from RII in the myocytes.
    RII, but not RI, was colocalized with AKAP100 in the rat heart.
    Our studies suggest that AKAP100 tethers **PKA** II to multiple subcellular compartments for phosphorylation of different pools of substrate proteins in the heart.

    - Cell Motil Cytoskeleton, 1994   **Regulation of motility and cytoskeletal organization of rat bladder carcinoma cells by cyclic AMP.** .
      Cyclic AMP cAMP has been implicated in the regulation of movement of certain cultured cell types.
      We have studied the effects of cAMP on epithelial cell motility using serum free NBT II cells, derived from a rat bladder carcinoma.
      The random movement of these cells on type I collagen was reduced upon elevation of intracellular cAMP by several means and this effect was reversible.
      Alterations in the organization of the cytoskeletal proteins F actin and alpha **actinin** occurred concurrently with the reduction in motility, and the arrangement of these proteins resembled that seen in non motile cells on glass.
      In addition, pretreatment of cells with KT5720, a cAMP dependent protein kinase **PKA** specific inhibitor, prevented the dibutyryl cAMP induced reduction in cell movement as well as the associated cytoskeletal changes.
      These results suggest that elevation of **PKA** is responsible for the observed effects on cell motility and cytoskeletal reorganization and demonstrate a role for **PKA** in the regulation of cell motility in this system.
